# Microbiota medicine: towards clinical revolution

**DOI:** 10.1186/s12967-022-03296-9

**Published:** 2022-03-07

**Authors:** Prisca Gebrayel, Carole Nicco, Souhaila Al Khodor, Jaroslaw Bilinski, Elisabetta Caselli, Elena M. Comelli, Markus Egert, Cristina Giaroni, Tomasz M. Karpinski, Igor Loniewski, Agata Mulak, Julie Reygner, Paulina Samczuk, Matteo Serino, Mariusz Sikora, Annalisa Terranegra, Marcin Ufnal, Romain Villeger, Chantal Pichon, Peter Konturek, Marvin Edeas

**Affiliations:** 1 International Society of Microbiota, Tokyo, Japan; 2 Department Endocrinology, Metabolism and Diabetes, Faculté de Médecine Cochin-Port Royal, Université de Paris, INSERM U1016, Institut Cochin, 24 Rue du Faubourg St Jacques, 75014 Paris, France; 3 Laboratory of Excellence GR-Ex, Paris, France; 4 Maternal and Child Health Department, Research Branch, Sidra Medicine, Doha, Qatar; 5 Medical University of Warsaw, Warsaw, Poland; 6 University of Ferrara, Ferrara, Italy; 7 University of Toronto, Toronto, Canada; 8 Furtwangen University, Furtwangen, Germany; 9 Department of Medicine and Surgery, University of Insubria, Varese, Italy; 10 Poznań University of Medical Sciences, Poznań, Poland; 11 Pomeranian Medical University, Szczecin, Poland; 12 Wroclaw Medical University, Wroclaw, Poland; 13 Université Paris Descartes, Paris, France; 14 Clinical Research Centre, Medical University of Bialystok, Bialystok, Poland; 15 IRSD, Université de Toulouse, INSERM, INRAE, ENVT, UPS, Toulouse, France; 16 National Institute of Geriatrics, Rheumatology and Rehabilitation, Warsaw, Poland; 17 University of Clermont Auvergne, Clermont-Ferrand, France; 18 Center for Molecular Biophysics CNRS UPR 4301, University of Orléans, Orléans, France; 19 Teaching Hospital of the University of Jena, Jena, Germany

**Keywords:** Dysbiosis, Built environment microbiome, Metabolites, miRNAs, Fecal microbiota transplant, Prebiotics, Probiotics, Oral microbiota, Metabolic syndrome

## Abstract

The human gastrointestinal tract is inhabited by the largest microbial community within the human body consisting of trillions of microbes called gut microbiota. The normal flora is the site of many physiological functions such as enhancing the host immunity, participating in the nutrient absorption and protecting the body against pathogenic microorganisms. Numerous investigations showed a bidirectional interplay between gut microbiota and many organs within the human body such as the intestines, the lungs, the brain, and the skin. Large body of evidence demonstrated, more than a decade ago, that the gut microbial alteration is a key factor in the pathogenesis of many local and systemic disorders. In this regard, a deep understanding of the mechanisms involved in the gut microbial symbiosis/dysbiosis is crucial for the clinical and health field. We review the most recent studies on the involvement of gut microbiota in the pathogenesis of many diseases. We also elaborate the different strategies used to manipulate the gut microbiota in the prevention and treatment of disorders. The future of medicine is strongly related to the quality of our microbiota. Targeting microbiota dysbiosis will be a huge challenge.

## Background

Microbial medicine has evolved thanks to the tremendous improvement in the understanding of genomics, metagenomics, and metabolomics in the recent years. In light of these advances, modulation of the host microbiome has been proposed as a potential treatment or prophylaxis for many health disorders. In fact, the human body harbors a huge array of microorganisms, among which bacteria have a great role. Other microbes also inhabit our bodies such as viruses, parasites, and fungi [1]. Together, these microbial communities form the human microbiota found in many areas within the human body, such as the skin, the upper airways, the gut, and the genital tracts [2]. The gut saprophytic commensal flora plays a fundamental role in the modulation of several local functions including nutrient absorption [3], the regulation of host immune system [4] and the defense against pathogenic microorganisms [5]. The gut microbiota is very diverse and its density changes along the gastrointestinal tract. However, its diversity is easily altered by different exo- and endogenous factors such as drugs, diet, health status, hygiene and surrounding environmental microorganisms [6]. Alterations in the symbiotic relationship between the microbiota and the enteric microenvironment, comprising cells of the innate and acquired immune system and enteric neurons, underlay development of complex gut disorders, including diarrhea [7], and chronic inflammatory bowel disease (IBD) [8, 9]. Gut microbiota dysbiosis is also involved in many systemic metabolic diseases [10] and neurological disorders [11]. Due to its importance in the pathogenesis, diagnosis, and treatment of many diseases, the mechanistic understanding of the gut microbiota diversity and metabolites became increasingly very important in medicine.

Even though we are still in the basal level of understanding the mechanisms involved in the cross-talk between the microbiota and the surrounding host environment, developing new therapeutic strategies to manipulate the gut microbiota has emerged as an evolving need in medicine, due to the important role of these microorganisms in the onset and the progression of many distant and local diseases.

In this review, we summarize the most recent and important advances in the discovery of the gut microbiota role in human health and diseases. The first part of our review focuses on the external factors that affect the human microbiome, known as the built environment microbiome in this case. The following parts highlight the involvement of the gastrointestinal microbiota in the pathogenesis of many diseases on many axes, such as the gut-lung, gut-brain axes and others. Finally, we summarize the recently discovered strategies to handle the gut microbiota in the prevention and treatment of various disorders.

## Human microbiome and microbiota dysbiosis in human diseases

**Fig. 1:**
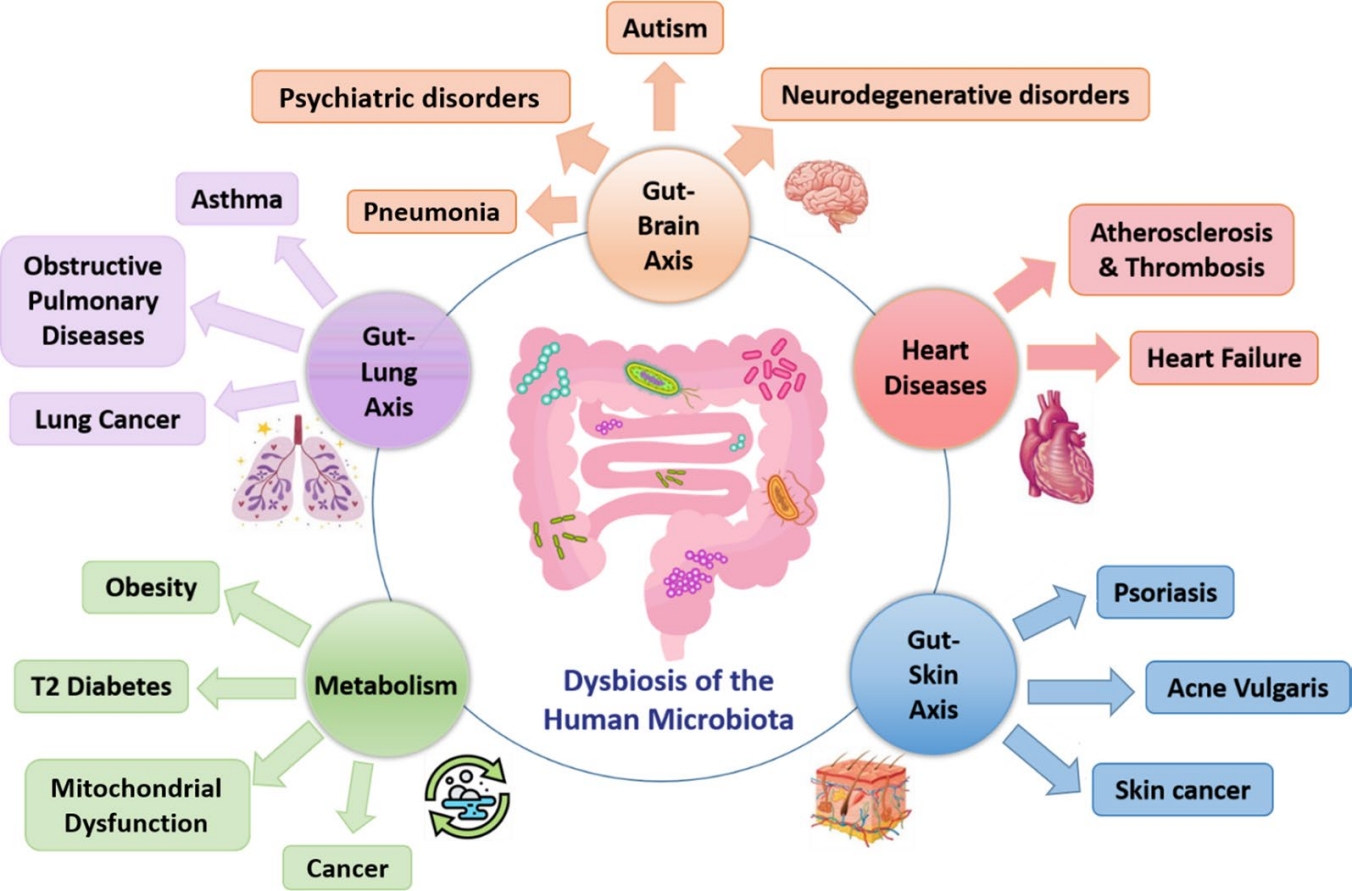
The human microbial dysbiosis in human diseases. Gut microbiota is implicated in the right functioning of many organs, such as lungs, kidneys, liver, heart and brain. However, any disruption to the microbiota homeostasis results in the malfunctioning of these affected organs, and the progression of many related diseases

Recent advances in the study of the human microbiome have improved our knowledge of all normal microbial communities that belong to the human body. So far, microbiota studies revealed that microbe host interactions exist not only within an organ, but also constitute an inter-kingdom crosstalk linking automatically distinct organs together. Indeed, the gut microbiota was extensively investigated, and was shown to be involved in the regulation of homeostasis in many organs including the gastrointestinal tract, locally, and the lungs and brain, systematically. Furthermore, gut dysbiosis plays a role in the progression of many diseases through the most important inter-organ connections such as gut-lung and gut-brain axes (Fig. 1).

### Built environment microbiome

The human microbiome is in a constant and dynamic interaction with the surrounding environment. It is affected by many factors involved in the daily routine of individuals such as the location, dietary intake, pollution and others. Indeed, people in urban life spend most of their time in enclosed buildings designed, built and managed by humans. For instance, in industrialized countries people spend up to 90% of their lifetime indoors, i.e. inside the built environment (BE) [12, 13]. The BE can be defined as “man-made structures, features, and facilities viewed collectively as an environment in which people live, work, recreate and travel” [13]. It comprises all structures built by humans including residential houses, public buildings, industry facilities, transportation vehicles, open spaces, but also very extreme environments, such as submarines and space stations [14]. Like in every ecosystem on the surface of the earth, different microbial communities have been found in every part of the BE and have been known as the “built environment microbiome”.

The BE microbiome and its interactions with human occupants represent a relatively new area of study and a highly interdisciplinary research field. The BE is characterized by a great microbial diversity, as well as a very fluctuating environmental conditions and sharp gradients of physicochemical parameters, which significantly shape the resident microbiomes [12]. Indeed, the composition and function of microbial communities found in the BE as well as their interaction with humans are extraordinarily complex, dynamic, and highly interconnected. Notably, such microbial’s communities’ microbes and their metabolites have been linked to the cause, exacerbation, or prevention of inflammation and other human diseases.

Due to a significant exchange between the human microbiome, in particular the skin and intestinal tract, and the BE microbiome, the essential role of the BE microbiome is evident but far from fully understood [14]. A depletion of microbial diversity in the BE is supposed to be partly responsible for an increasing prevalence of allergy and asthma in industrialized countries. In addition, there is mounting evidence that the man-made environmental conditions inside the BE, such as the extensive use of antimicrobial cleaning agents, may favor polyextremophilic microorganisms that pose a threat to the human inhabitants [15]. Nevertheless, the control of pathogenic microorganisms in the BE clearly requires antimicrobial measures to prevent infections [13].

Similarly, considerable efforts have been made to understand the effect of the BE and its microbiome on the human mental health [16, 17]. Indeed, with the increased trendy urbanization, people exhibit limited exposure to the so-called immunoregulatory microbes, referred to as the “old” friends. Such a decrease in exposure has led to an epidemic of chronic low-grade inflammatory states associated with an increased risk of stress-related psychiatric disorders [18].

Probably, a successful management of the BE microbiome for the sake of human health will require a well-balanced mixture of, among others, antimicrobial and “probiotic” measures [13, 19, 20].

### Oral and intestinal microbiota dysbiosis in human diseases

The digestive tract constitutes the largest interface between the sterile part of the human body and environmental factors and pathogens. It is the most important site for colonization by thousands of microorganisms such as viruses, eukaryote, and more than 1000 types of bacteria. These microbes, named collectively "gut microbiota", are well-known to have different beneficial roles in maintaining the human homeostasis such as strengthening gut integrity, harvesting energy, protecting against pathogens, and regulating host immunity [21].

In fact, humans first encounter microorganisms in their early life, specifically at birth when different bacteria succeed to colonize the baby’s body during the first months of life. In details, microorganism start to develop in the oral and nasopharyngeal membranes as well as the skin. Gradually, the microbiota will start to increase steadily within the gastrointestinal tract, with a developed oral and salivary microbiome, less bacteria in the stomach, but with a very high concentration of bacteria inhabiting the colon [22].

A tremendous amount of extensive research work has been done in the last decades, and has revealed that the gut microbiota dysbiosis can be associated with many pathologies within the human body [21], such as periodontitis and caries [23, 24], various metabolic disorders [25–27], chronic inflammatory bowel syndromes [28], cardiovascular diseases (CVD) [29, 30], cancer [31, 32], as well as chronic kidney diseases (CKD) [33, 34]. The extent to which gut microbiota dysbiosis may exert this systemic control and cause the induction of a given pathology depends on the functionality of gut barrier and the maturity of the immune system of the recipient [35].

#### Oral and gut microbiota dysbiosis in carcinogenesis

Daily changes of the oral microbiota and the salivary cytokines in healthy individuals were recently linked to the circadian oscillations [36]. The recent characterization of the salivary microbiome revealed that the diversity of the oral and salivary microbiota is affected by the age, the oral health, denture use, smoking and coffee-tea consumption [37]. Moreover, the alteration of both structure and function of gut microbiota has systemic consequences, widely beyond those related to digestion [38].

In fact, it is estimated that microorganisms could be associated with tumorigenesis in 15% to 20% of cancers, which represent the second leading cause of mortality worldwide. Correlative studies using 16S rRNA sequencing have associated gut bacterial communities with pathologic conditions such as cancer. This suggests that a disruption of the intestinal microbial homeostasis may affect the host responses in the maintenance of health. Increasing evidence suggests that the human commensal microbiome is involved in the etiopathogenesis of cancers [39], but to date, finding a proof of causality is still a major challenge in this field. Gut microbiota was not only able to affect colorectal carcinogenesis [40, 41], but it also seemed to affect other types of cancer distant from the gut such as lung [42] and prostate carcinomas [43]. Since several studies have demonstrated the role of the gut microbiome in the resistance to therapeutic strategies including surgery, chemotherapy, radiotherapy and immunotherapy, it is vital to understand the specific role of microbes in cancer development and treatment efficacy to improve patient’s outcome [44]. Among these gut microbes, the most known bacterial carcinogen is *Helicobacter pylori* [45]. Several studies have also reported that genotoxic colibactin-producing *E. coli* (CopEC) [46] are associated with the development colorectal cancer (CRC). The pro-carcinogenic effect of CopEC has been confirmed In-vitro and In-vivo [47, 48]. Interestingly, CopEC are preferentially detected in patients with CRC and are more prevalent in the mucosa of patients presenting advanced stage III/IV CRC than in those with stage I CRC [49, 50]. Moreover, CopEC have been demonstrated to modulate the response to cancer immune checkpoint inhibitor [51]. These data suggest a promising use of CopEC as a prognostic factor in CRC. Overall, the use of microbial markers not only for cancer prognosis, but also as possible targets for therapeutic intervention seems promising. However, it requires the development of fast, robust and standardized screening methods along with longitudinal international cohort studies. Moreover, some periodontal bacteria have been recently studied and appeared to be associated at the center of the link between the oral microbiota dysbiosis and cancer. In this group, the carcinogens mentioned most often are *Peptostreptococcus sp.*, *Prevotella sp.*, *Fusobacterium sp.*, *Porphyromonas gingivalis*, and *Capnocytophaga gingivalis*. Many works have also shown that *Fusobacterium nucleatum* and *Porphyromonas gingivalis* play an important role in the development of colorectal and pancreatic cancer. Three mechanisms of action have been suggested in the pathogenesis of cancer by oral microbiota [52, 53]:

- Bacterial stimulation of chronic inflammation (by induction of IL-1β, IL-6, IL-17, IL-23, TNF-α, MMP-8 and MMP-9),
- Influence on cell proliferation (by activation of antiapoptotic Jak1/Akt/Stat3 signaling, manipulation of cyclin/CDK activity, reduction of the p53 level, etc.),
- Production of carcinogenic substances (reactive oxygen species, reactive nitrogen species RNS, volatile sulfur compounds and organic acids).

#### Microbiota dysbiosis in metabolic syndromes

Recent studies suggest that gut microbiota dysbiosis is associated with many metabolic disorders such as obesity, hyperglycemia, dyslipidemia, and others. Such studies made it clear that the gut microbial dysbiosis is involved in the regulation of fat storage within the human body, and consequently the occurrence of obesity [54]. Indeed, an increased dietary fat intake, typical in Western countries, is one the strongest triggers of gut microbiota dysbiosis, as well as a known inducer of metabolic diseases such as obesity and type 2 diabetes. Of note, this alimentary fat excess, along with a high sugar intake, were proven to be some of the main factors prompting colonization of intestine by enterobacteria [55], like the pro-inflammatory and/or genotoxic *Escherichia coli*. These bacteria may affect the stability of host cells’ DNA via the production of colibactin, a genotoxin shown to induce DNA double strand breaks [46], thus exerting a pro-tumorigenic activity [47]. Very recently, colibactin has been shown to affect the evolution of both gut microbiota and microbiome of mice progeny, whose mothers were infected with genotoxic SP15 *E. coli*. [56]. Therefore, based on these evidences, gut microbes appear to be at the center of a new metabo-infectious triad among gut microbiota dysbiosis, metabolic diseases and enterobacterial infections. Nonetheless, a great body of previous reports revealed the involvement of gut microbiota in the development and the progression of type 2 diabetes (T2D). The most common and recent reports reviewed the prevalence of *Bifidobacterium*, *Bacteroides*, *Faecalibacterium*, *Akkermansia* and *Roseburia* in the pathogenesis of T2D [57].

#### Microbiota dysbiosis in chronic kidney diseases

A substantial body of literature has provided evidence on the presence of a bidirectional interplay between the gut microbiota dysbiosis and the chronic inflammation within the human body, whether it is local or beyond the gastrointestinal tract. It was shown that changes in the composition and function of the microorganisms in the gut are associated with a systemic inflammatory state and play a role in the development of the CKD [33, 58]. In detail, a recent study done by Li Y. et al. revealed that patients diagnosed with stage 5 CKD, compared to healthy individuals have a significantly higher abundance of *Neisseria*, *Lachnoclostridium* and *Bifidobacterium*, and a lower abundance of *Faecalibacterium* [59]. Another recent research study showed, for the first time, that there is a decrease in the abundance of *Akkermansia*, an important probiotic, in CKD patients. Nonetheless, altered levels of many bacterial genera were associated with abnormal severe indicators such as the low glomerular filtration rate and high secretion of Interleukin-10 [60]. In fact, not surprisingly, the gut dysbiosis is not only involved in the development of CKD, but it also appears to be a novel and main risk factor in the worsening of the disease and the progression of its complications such as cardio-vascular comorbidities. Indeed, it was reviewed that an alteration in the gut microbiome is involved in many hypertensive effects in rat models and humans, as well as in increasing the bile acids levels in blood which are directly linked to a high risk of CVD [61]. External factors are also able to worsen the gut dysbiosis, and, thus, lead to a fast progression of CKD complications. Among such factors, different studies revealed that hemodialysis could exacerbate the gut microbiome dysbiosis. In details, a recent study by Durand et al. revealed that the gut microbiota diversity seemed lower in hemodialysis patients under citrate dialysate. Cramps and over-expression of some bacterial genera like *Helicobacter*, *Lachnospira*, *Roseburia*, and *Haemophilus* were also reported. Significant citratemia and lower mitochondrial functions were also observed in such patients [62]. Animal models demonstrated the efficacy of prebiotics intervention improving the gut microbiota in CVD, opening the route to new therapeutic approaches including microbiota modulators [63].

#### Microbiota dysbiosis in chronic liver diseases

Gut microbiota communicate with the liver, through different complex pathways. Therefore, any change in the gut microbiota can strongly affect the liver. Gut dysbiosis, caused by altered intestinal permeability and damaged bile acid metabolism, could reach the liver and lead to systemic inflammation. These alterations are different from a disease to another, and it has been proven that the severity and type of chronic liver disease strongly depend on the progression of gut dysbiosis [64]. Several liver chronic diseases result from such alterations: as chronic hepatitis B, chronic hepatitis C, alcoholic liver disease, non-alcoholic fatty liver disease, non-alcoholic steatohepatitis (NASH), liver cirrhosis, and hepatocellular carcinoma [65].

One of the examples is NASH which is characterized by a buildup of fat in the liver and is typical in patients with obesity and excess of fat. Some studies showed that patients with NASH had increased amount of *Firmicutes*, with a decreased quantity of *Bacteroidetes* [66]*.* In these chronic liver diseases, pathogen-associated molecular patterns (PAMPs) are more exposed to oxidative stress, leading to further inflammation by the production of cytokines through TLRs. Another key player in these diseases is the bile acid. The latter is essential for fat solubilization. Due to microbiota dysbiosis, bile salts are preserved leading to the permeability of gut microbiota and bacterial growth. All of these consequences are in favor with liver disease [65, 66].

#### Microbiota dysbiosis and physiological changes

The diversity and the functions of the gut microbiota were shown to be affected by naturally occurring physiological changes that accompany pregnancy, including the immunological and hormonal gestational balance. In fact, the estrogen and progesterone affect the prenatal and postpartum intestinal motility in women as well as the gut microbial diversity through their effect on bacterial metabolism, growth and virulence [67]. In one of the most recent cohort studies, it was demonstrated that the gut bacterial repertoires largely differ in pregnant women under the effect of multiple factors such as gestational age, body mass index, ethnicity, nutritional state and others [68]. The effect of the gut microbiota dysbiosis throughout the different pregnancy trimesters has been of special concern as it may significantly contribute to different metabolic disorders during the pregnancy stages [69]. Recent evidence demonstrated a connection between gut microbiome and gestational diabetes mellitus (GDM) referred to as a glucose intolerance in pregnancy, at the first [70], second [71] and the third trimester of pregnancy [72]. It was also shown, in a recent study, that GDM pregnant women show special food preferences such as lower intake of vegetables, fish, poultry, and fish paste. This correlates with microbiome diversity [73]. In this context, the use of probiotics as a novel therapeutic strategy appears to be appealing in the prevention of type 2 diabetes mellitus in post-GDM women. The consumption of multi-strain probiotics can actually contribute to the modulation of the gut microbiota composition, the improvement of the intestinal epithelial integration and others. Further investigations on the probiotics selection, the dosage, the timing and the duration are needed [74].

Nonetheless, the imbalance in the gut microbiota during pregnancy is associated to pre-eclampsia (PE) which is a pregnancy-specific systemic disorder involving hypertension, proteinuria and other complications [75]. One recent case–control study revealed a reduction in the bacterial diversity. The gut microbial community was enriched by opportunistic pathogens, such as *Fusobacterium* and *Veillonella*, but lacked the beneficial bacteria. This striking dysbiosis was correlated to an increase in the blood pressure, as well as in the proteinuria, aminotransferase and creatinine levels. Moreover, the T regulatory/ helper -17 cells balance was greatly disturbed in the intestines and the spleen of mice with transplanted fecal microbiota from PE patients. However, the role of gut microbiome in the pathogenesis of PE remains not fully elucidated and requires lot of intensive studies [76]. Therefore, it can be concluded that the gut microbiome can potentially be used as an early biomarker of pregnancy complications, such as GDM and PE.

Among hormonal women pathologies, endometriosis is a chronic, estrogen-dependent, benign disease characterized by the presence of endometrial tissue outside the uterus. It affects 6–10% of women in their reproductive years, causing chronic pelvic pain and infertility. Its pathogenesis remains poorly understood and current treatments, based on hormonal therapy or surgery, are often insufficient. Treatment with one or two probiotics, have different but both favorable effects on clinical, immune and physiologic parameters in endometriosis. Because of its better results on pain and a greater ease of handling, *Saccharomyces boulardii* seems to be more suitable to be used as a new therapeutic strategy for endometriosis [77].

#### Microbiota-drug interaction and psychiatric disorders

The gut microbial dysbiosis has been also described upon the use of many drugs in the treatment of a wide variety of diseases, either locally or away from the digestive tract. Indeed, not only the traditional antibiotics, but also other drugs and bioactive molecules have been shown to have an antimicrobial activity [78]. Some of these drugs include the ones that are used in the treatment of psychiatric disorders. The recurrent use of these medications can be of significant importance for patients. Commonly used antidepressants differ in mechanisms of their antibacterial activity; for example, Monoamine Oxidase Inhibitors can disturb bacterial cell-wall synthesis, tricyclic antidepressants (TCAs) can inhibit DNA gyrase activity and plasmid DNA replication, and selective serotonin reuptake inhibitors can inhibit bacterial efflux pumps [79]. In a recent study, it was revealed that a 6-week escitalopram treatment in a psychiatric hospital setting resulted in increased alpha biodiversity (richness and evenness) in fecal microbiota, highlighting the antibacterial activity of this drug [80]. Results of another study shown that gut microbiota was associated with the severity of depressive symptoms, but is not a predictor of antidepressant treatment efficacy. Interesting that intestinal barrier integrity and inflammation markers were associated with the response to treatment of patients with major depressive disorder (MDD) [81]. However, it should be stressed that the causal relationship between microbiota and MDD has not yet been unequivocally confirmed [82]. Recent meta-analysis reported that psychobiotics (probiotics improving mental health) are promising in the treatment of MDD. However, no specific strain/strains, dosage or duration of treatment can currently be recommended [83]. Antipsychotic treatment-related microbiome alterations potentially result in body weight gain and metabolic disturbances. Inflammation and resting metabolic rate suppression caused by Second Generation Antipsychotic Drugs seem to play crucial roles in the development of metabolic disorders [84]. However, this is still controversial, since it was found, in another study, that the microbiota of schizophrenia patients is highly individual but can be divided into different taxonomical and functional clusters and it does not change following six weeks of olanzapine therapy. In another work, microbiota disturbances did not affect neither the weight gain observed in women nor the effectiveness of olanzapine therapy [85]. Until now, the knowledge in this field is still very limited for practical applications and drugs—microbiota interactions surely require more studies. They should be considered especially in long term treatment strategies for mental disorders.

### Gut microbial metabolites in different disorders

Although research in the past decades showed a bidirectional crosstalk between host and microbiota, the mechanisms in this interaction are not fully described. Interestingly, recent studies demonstrate that not only the nature of the gut microbes and their diversity influence the host, but also their metabolites [86]. The gut microbiome yields a myriad of metabolites involved in the regulation of immune functions, as well as metabolic, and neuronal responses at local and distant sites [87, 88]. Indeed, the gut microbial metabolites are intimately linked with the host health and diseases. Emerging metabolite-centered research has focused on identifying different actionable microbial targets, whether intermediate metabolites or by-products, that are relevant in many host diseases such as metabolic syndrome [89], obesity [90], diabetes [90, 91], cardiovascular morbidities [92, 93], and even cancer [94–96].

#### Metabolites in cardiovascular diseases

Among gut bacterial metabolites that affect systemic functions such as the circulatory system, methylamines, namely trimethylamine N-oxide (TMAO) and trimethylamine (TMA), have gained the greatest attention. TMA is produced by several bacterial genera from carnitine and choline and after passing gut-blood barrier it is oxidized in liver to TMAO. Recently, the positive correlation between plasma TMAO concentration and cardiovascular risk has been shown [97–99]. It is suggested that plasma TMAO may be used in risk assessment in general population and in patients with CVD like heart failure, coronary artery disease, peripheral artery disease etc. Therefore, many studies attempted to establish the causative role of TMAO in cardiovascular pathologies. However, the data are inconsistent and it is still not clear whether it is a harmful compound, a component of adaptive response or only a confounder. Surprisingly, TMA, which is a noxious precursor of TMAO, have not been sufficiently investigated. Toxic properties of TMA including eye and skin irritation, developmental damage, behavioral disorders and encephalopathy have been described in medical literature nearly 100 years ago and the metabolite is considered as a uremic toxin. However, its role in circulatory system have not been established. Recently, it was demonstrated that TMA, but not TMAO, elevates blood pressure [97], exerts cytotoxic effect on cardiomyocytes and is responsible for increased mortality rate [98]. It was also observed that TMA increases with age and affects vascular smooth muscle cells viability [99]. These findings suggest that both methylamines should be measured with regards to cardiovascular risk assessment.

#### Metabolites in cancer

Intriguingly, recent works revealed that the relation between microbiota and cancer is mediated by microbial metabolites [94]. Indeed, mechanisms of some well-known bacterial genotoxins and oncogenes, such as colibactin, in the tumor progression are now well described. It was shown that such metabolites are thought to be involved in the alterations of the cell cycle and in the regulation of the immune response through transcriptional and epigenetic regulation [100]. Most of the metabolites, analyzed in literature, presented a double effect being either promoters or suppressors of carcinogenesis. However, the only metabolites that have been purely considered as anti-carcinogenic are short-chain fatty acids [101] and polyphenols metabolites [96, 102].

Moreover, the gastrointestinal microbial metabolites and their interplay with the host are not only affected by endogenous changes, but also by external factors. Indeed, external interventions such as gastric surgeries can have their own impact on the gut microbiome and metabolites [103]. Modified levels of carnitines, lipids, amino acids (including Branched Chain Amino Acids) and α- and β-hydroxybutyric acids were detected in T2DM patients who underwent laparoscopic Roux-en-Y gastric bypass or laparoscopic sleeve gastrectomy. Some of the observed metabolites suggest that changes in gut microbiota composition may also correlate with the pace of diabetes recovery. Additional analyses confirmed a relationship between biochemical and clinical parameters and the aforementioned metabolites, thereby, highlighting the role of mitochondria and microbes in observed metabolic and clinical effects of surgeries [104–106].

### Gut-lung axis

Even though these organs are anatomically distant from each other, the microbial kingdoms at the respiratory and digestive tracts have maintained a dynamic interaction that play key roles in the body homeostasis and in disease evolution [107]. From birth till death, a close relation links the microbial community in the lungs to the gut microbiota, suggesting a microbe-microbe interaction as well as a microbe-host interplay. For example, any change in the neonate’s diet is able to affect the respiratory microbiota composition [108]. Moreover, a recent study showed that fecal microbial transplant can alleviate the acute pulmonary injury in rats, by reducing the inflammatory factors’ expression [109] and possibly can reduce the risk of COVID-19 progression to the more advanced stages [110].

In fact, mounting evidence suggests that the gut-lung axis is a central element connecting microbial dysbiosis to many human diseases. Hosts either lacking gut microbiota, i.e. germ-free mice, or treated by broad spectrum antibiotics exhibited low or impaired immune responses against viral infections such as influenza Virus or respiratory syncytial virus, as demonstrated by increasing number of research [111–113]. In the same context, many clinical issues noticed that malnutrition and microbial dysbiosis in the gut are likely to play an important role in the progression of age-related respiratory diseases in elderly. These findings suggest that restoring an eubiosis of intestinal microbial community will promote a healthy aging [114].

The perturbation of the microbial composition and function is not only behind the increased risk of pulmonary infections, but it is also associated with tissue disruption and inflammatory diseases in the gastrointestinal tract and beyond. Higher incidence of pulmonary functional changes and diseases are found in patients with IBD, as compared to normal people [115, 116]. Moreover, the impairment in the gut-lung axis was shown to be associated with increased prevalence of asthma [117, 118]. Commensal microbes are necessary especially during early life, for the induction of a balanced, tolerogenic immune system. The disturbance of such microbiota composition in the gut-lung axis appeared to be associated with the onset and severity of the inflammatory mechanisms of the atopic asthma [119]. Efforts were made in a trial to reconstitute the microbiota, mainly in the gut, either by probiotics or engineered bacteria in order to reduce asthma progression [120]. Indeed, studies showed that early supplementation by *Lactobacilli* can decrease the risk of asthma in childhood [121].

Nonetheless, emerging pathogenic links between the gut-lung axis and the altered microbiota suggests that such dysbiosis might be important in the development of chronic obstructive pulmonary disease (COPD) [122]. A bidirectional interaction is responsible for the development of COPD in patients with IBD (i.e. ulcerative colitis) [123], and vice versa [124]. As such, the diet of COPD patients is of great importance and can be considered as one of the factors that exacerbate the COPD. Targeted dietary intervention by increasing the fiber intake increased the production of anti-inflammatory short chain fatty acid (SCFAs), and thus reduces the lung inflammation in COPD patients [125]. Additionally, owing to its power in maintaining the lung homeostasis, any dysfunction in the lung-gut axis and its respective microbiota could be critical in tumorigenesis and induction of lung cancer. However, their exact role in cancerogenesis and the potential therapeutic strategies to alleviate the cancer remain unclear [126, 127].

Finally, in light of the striking COVID-19 pandemic, decreased richness and diversity of the gut microbiota was correlated with immune deregulation, prolonged diarrhea, delayed viral clearance, and even with increased mortality by exacerbating lung infections. Thus, targeted probiotics treatment may be useful in the re-establishment of the microbial communities in the mucosal compartments of both GI and respiratory tracts and could be considered as adjunctive therapy in COVID-19 patients [128, 129].

### Gut-brain axis

As previously discussed in the above parts, the symbiotic gut microbiota is involved in the regulation of many homeostatic mechanisms within the body. The alteration of such intestinal microbial community was indeed associated with many metabolic, immune, and neurological disorders [21]. The relationship between the intestinal microbial diversity and the brain function has recently gained the attention of the scientific and medical community. Recent and increasing body of evidence suggest the presence of “two-ways” interaction between the gut microbiota and the brain, involving multiple neurological and endocrine signaling systems. The discovery of a such bidirectional cross-talk is reflected by the term ‘brain-gut-microbiota axis’ [130]. Accumulating experimental and clinical data confirm a key role of gut dysbiosis and disturbances within the brain–gut–microbiota axis in neurodegenerative processes [131–134] such as stress-related disorders [135], autism spectrum disorder [136], schizophrenia [137], multiple sclerosis [138], Alzheimer’s disease (AD) [139], and Parkinson’s disease (PD) [140].

For instance, an induced condition of chronic stressful period in mice has altered the gut microbiota diversity with a reduced amount of beneficial bacteria such as *Lactobacillus*, *Eubacterium rectale*, *Lachnospira*, along with an increase in the number of pathogenic bacteria like *Clostridium*, *Enterobacteriaceae*, and others [141].

Moreover, it is becoming increasingly evident that the gut microbiota influences the central nervous system’s function and inflammation through different pathways. Among the latter, the innate immune inflammasome signaling complexes are overactivated when the gut microbiota is altered with presence of certain infectious agents or pathogenic intestinal bacteria. This over-activation of inflammasomes in the brain has recently been associated with the development and the progression of many neuroinflammatory conditions such as multiple sclerosis, anxiety, as well as Parkinson’s and Alzheimer’s diseases [142]. Nonetheless, the two most common neurodegenerative disorders –AD and PD—are characterized by proteopathy: amyloid beta (Aβ) deposition in AD and α-synucleinopathy in PD. The formation of abnormal protein aggregates takes place both in the enteric and central nervous systems. A large amount of amyloids is also secreted by the gut microbiota. Through molecular mimicry bacterial amyloids may cross-react with human antigens and elicit cross-seeding of misfolding inducing disturbances in the adaptive immune system and microglial priming. A new term “MAPraNosis” has been even proposed to describe the process of microbiota-associated proteopathy and neuroinflammation [143]. Moreover, age-related alterations in the gut microbiota composition characterized by its decreased diversity and stability are associated with so called “inflammaging” resulting in the immune system activation in the elderly, and disruption of the intestinal barrier and the blood–brain barrier inducing neuroinflammation and neurodegeneration [144]. There is a growing interest in the prion hypothesis of neurodegenerative disorders according to which the neurodegenerative cascade may be initiated in the gut with subsequent spreading of proteopathy including Aβ and α-synuclein aggregates from the gut to the brain in a prion-like manner [131, 132]. In this context, in one of the recent studies for Pasinetti et al., it was evident that the use of ketamine, a potential therapeutic drug in neuroinflammatory disorders, is associated with an increase in the number of beneficial intestinal bacteria along with a reduction in the opportunistic pathogens. This study opens the door for understanding the long-term safety of ketamine in the treatment of AD [139]. In another work, bacterial DNA could be isolated from the human brain suggesting that a human microbiome can exist [145]. However, further studies are needed to understand the difference in the human brain microbiome among normal individuals and patients diagnosed with AD since most of the studies are done on animal models. The impact of the gut microbiota on the development of the pathogenesis in the neurological disorders is, unfortunately, not fully understood.

The gut microbiota modulation with dietary interventions, probiotics or antibiotics may create new preventive and therapeutic options in neurodegenerative diseases [146]. Indeed, we already shown that dietary polyphenols and other probiotics can be used as novel therapeutic drugs in the treatment of depression and other neurological problems [96, 147]. Actually, these symbiotics were shown to be able to attenuate the neuroinflammation by decreasing the activity of the inflammasomes mentioned previously [145].

### Gut-skin axis

The skin, the largest organ of our body, is the outermost barrier of the organism. It protects us from external harm and senses danger signals. It contains a sophisticated immune system that involves armada of immune cells comprising keratinocytes, resident antigen presenting cell, innate lymphoid cells, innate-like cells, and adaptive tissue resident memory cells and molecular mediators [148]. The skin also hosts microbial communities known as skin microbiota, constituting one living response barrier to environmental factors. Those microorganisms live in complete harmony with the immune players and are involved in the epithelial barrier reinforcement. Under homeostasis, an established alliance of immunity and microbiota intermingles innate and adaptive branches of the immune system. Under various types of stress, the symbiotic relationship changes into a dysbiotic one resulting in skin and distinct organs pathologies [149]. Surprisingly, an increasing body of evidence revealed that skin pathologies are not only due to skin microbiota dysbiosis, but they are also accompanied with alterations within the gut microbiome. In fact, both the intestinal tract and skin are densely vascularized and extensively inhabited by a wide variety of microorganisms that play many roles in maintaining the homeostasis. Different studies demonstrated a bidirectional cross-talk between skin and gut microbiota, referred to as the gut-skin-microbiota axis [150–152]. This intimate connection, if altered, was shown to be associated with many skin diseases such as psoriasis [153], atopic dermatitis [154], and other disorders including skin cancer [155].

That so-called “gut-skin axis” has been considered as a key factor in the etiology of psoriasis, which is a chronic inflammatory disease with a complex multifactorial pathogenesis. The psoriatic skin and gut microbiota are characterized by a decreased overall diversity with a high prevalence of *Staphylococcus* and *Streptococcus* species on the skin [156] as well as *Bacteroidetes* and *Firmicutes* in the gut [157]. An increasing number of studies showed that the intestinal health as well as the individual lifestyle (such as smoking, alcohol, and increased body weight) play a pivotal role in the onset and the exacerbation of the psoriatic lesions [158, 159]. These facts have opened the door to target the dysbiotic gut microbiota in psoriatic patients by the use of probiotics [160]. Moreover, the gut-skin dysbiosis in psoriasis leads to intestinal barrier damage, which if intensified by the local inflammation, will increase translocation of antigens, bacteria and their metabolites into the circulation. Indeed, psoriatic patients exhibit very high blood concentration of TMAO and other gut microbiota-derived metabolites. This condition will put the patients with psoriasis at a high risk of CVD [161]. The intestinal inflammation in such patients is combined with an increased prevalence of IBD [162].

Another skin inflammatory condition which is affected by the gut-skin microbiota dysbiosis is the acne vulgaris. Acne is characterized by the formation of inflamed sebaceous sties, and affect around 90% of teenagers. Although the exact cause of acne is not fully elucidated, alteration of the skin microbiota as well as the host immune dysregulation play a role in the progression of such disease. Moreover, it is now believed that not only the skin microbiota but also the alteration in gut microbial communities is involved in the exacerbation of acne. For instance, the reason behind stress-induced acne is explained by the effect of the stress on the gastrointestinal tract. Stress increases intestinal permeability which leads to skin inflammation. Hence, there is an axis, involving the gut-brain-skin microbiota, that connects gut microbes, oral probiotics, diet as well as emotional and hormonal imbalance and contributes to the severity of acne [163].

## Therapeutic strategies in the gut microbiota manipulation

It is well-known that the gut microbiota not only plays great roles in the modulation of many physiological functions within the host body, but also its dysbiosis is involved in the pathogenesis of many local and distant disorders. Owing to this fact, developing new therapeutic strategies that can target the gut microbial communities became very interesting to establish personalized manipulation and treatment of many diseases. To achieve this goal, many strategies have been successful including prebiotics, probiotics, fecal microbiota transplantation, phages, and the emerging miRNAs.

### Prebiotics

Prebiotics are a group of nutrients that are degraded by gut microbiota. Glenn Gibson and Marcel Roberfroid first introduced the concept of prebiotics in 1995, being “non-digestible food ingredient that beneficially affects the host by selectively stimulating the growth and/or activity of one or a limited number of bacteria in the colon, and thus improves host health” [164]. The most recent definition of prebiotics is now “a selectively fermented ingredient that results in specific changes in the composition and/or activity of the gastrointestinal microbiota, thus conferring benefit(s) upon host health” [165]. They are classified into many types and the byproducts of their degradation by the gut microbiota are most of all SCFAs that can exert an anti-inflammatory effect in the regulation of many human disorders [166]. Owing to the SCFAs’ small size and ability to diffuse to the bloodstream, prebiotics could be thus an attractive treatment not only locally for the gastrointestinal tract, but also to other distant organ systems.

In fact, prebiotics are safe and effective, having a great therapeutic effect along with minimal side effects in the maintenance of IBD [167]. For example, in one of the earliest studies, Lindsay et al. showed that the use of 15 g of fructooligosaccharides in Crohn’s Disease patients had increased the mucosal *Bifidobacteria*, and reduced the inflammation index [168]. Moreover, prebiotics have been shown also effective against genetically-induced obesity at doses lower than the usually used grams per days and for a limited period of only 2 weeks [169]. Despite all the efforts, there are few studies on humans, and the majority of research work has investigated the efficacy of prebiotics in IBD in vitro or in animal models [170]. Prebiotics were also shown, alone or combined with probiotics, to positively influence the cross-talk between immune system and microbiota, in the prevention of the CRC in patients with its high risk [171]. Therefore, prebiotics intake is recommended to improve the immunological response in CRC patients either in their preoperative or postoperative periods, as shown in a recent study by Xie et al., confirming that prebiotics consumption could change the abundance of four commensal microbiota [172].

At distance from the gastrointestinal tract, these non-digestible oligosaccharides are proven to be effective in the modulation of the gut-brain axis, more specifically in the regulation of the neurological disorders [173]. Several studies showed that the use of prebiotics can improve the lifestyle of patients with Huntington’s disease, and can also alleviate the severity of symptoms [174, 175]. Moreover, prebiotics showed a good preventive effect in many of neuropsychiatric disorders such as autism and depression [176]. Indeed, many studies reveal mental health benefits of prebiotics in females by reducing the stress and anxiety in a matter of days [177].

### Probiotics

The clinical use of probiotics to modulate gut microbiota has been documented in the context of therapeutic trials of many diseases. In fact, probiotics, referred to as “live organisms that when administered in adequate doses confer a health benefit to the host” [178], are administered in food, pills, or powders, and are available in pharmacies, and even in the online shops. Probiotics have been successfully used in the prevention and treatment of obesity in infants and adults [179]. Such administration of beneficial microorganism was shown to reduce HbA1c and the insulin resistance level in T2 diabetes mellitus patients [180, 181]. The use of probiotics, whether single-strain or multi-strain, was studied in the treatment of irritable bowel syndrome [182]. In particular, the “next-generation” probiotic *Faecalibacterium prausnitzii* has promising effects in the gut dysbiosis diseases improvement. Lower amounts of *F. prausnitzii* were detected in IBD, *Clostridioides* (formerly *Clostridium) difficile* infection (CDI), and COVID-19. As this strain of bacteria is butyrate-producing, it induces immune responses and anti-inflammatory effects, as well as improved intestinal barrier function [183, 184].

The use of probiotics was not only advised in the use of gastrointestinal and metabolic diseases, but also interestingly in more distant disorders such as respiratory infections [185], and neuro-inflammatory pathologies [185] including multiple sclerosis [186] and AD [187].

Probiotics were also used to control the transmission of the nosocomial infections, referred to as healthcare–associated infections (HAIs). Major causes of HAIs include the persistent microbial contamination of the hospital environment, as well as the growing antimicrobial-resistance (AMR), due to the selective pressure exerted by the huge and extensive use of disinfectants and antibiotics in hospitals. To this aim, monocentric and multicenter studies were performed, by substituting the conventional chemical-based cleaning procedures with a probiotic-based system, and examining simultaneously the microbiome of the hospital environment, its antibiotic-resistant genes content, called ‘resistome’, the incidence of HAIs and the associated therapy costs. The results showed that probiotic-based sanitation system could induce a stable remodulation of the hospital microbiome, allowing a stable control of bioburden and AMR, and a significant reduction of HAI incidence, HAI-related antibiotic consumption and HAI-related therapy costs [188, 189].

### Fecal microbiota transplantation and fecal virome transplantation

The fecal microbiota transplantation (FMT) consists in the introduction of liquefied or encapsulated pre-processed stool from a healthy donor into a recipient’s colon. In fact, FMT (or fecal bacteriotherapy) became increasingly and rapidly accepted and medically performed because of its success in treating bacterial infections, mainly *C. difficile* [190], but also, intriguingly, in the treatment of other disorders like obesity and diabetes [191], as well as the metabolic syndrome [192].

Microbiota Transplantation is now recognized as an efficient therapeutic for the management of recurrent *Clostridioides difficile*-induced colitis, which is a major cause of nosocomial disease with increasing occurrence and mortality [193]. Such infection is usually associated with the repetitive use of antibiotics and its relapses are often seen in 20–30% of the patients after the first antibiotic treatment. The unusually high success rate of FMT (85–95%) in this setting is the reason why it is recommended by the European and North American medical professional societies in this setting [194]. However, patient access to FMT remains hampered by logistic aspects of treatment including manufacturing, storing, delivering the fecal inocula, as well as the protocols of preparation. Recent studies have reported the use of capsules of either frozen or freeze-dried stool allowing oral administration in the in- and outpatient settings, prepared by different protocols [195, 196]. Altogether, these studies represent an important step forward to improve the standardization of the stool preparation procedures and reduce the process time, thus facilitating pharmaceutical and medical practices and comfort of patients.

Nowadays, a growing interest for FMT treatment is rising, because intestinal diseases other than infection due to *C. difficile* may benefit from this practice [194, 197]. However, clinical trials have yielded mitigate results highlighting an important donor stool effect. The new goal is thus to refine donor’s selection beyond safety to optimize the quality and efficacy of this new treatment beyond rCDIs. In this regard, animal models still remain crucial for the development and a better understanding of how FMT works on both donor and recipient side. In fact, also the preparation of the recipient may account for the efficacy of FMT. FMT without antibiotics and laxatives preparation before the procedure in the recipient showed lower success rates [35].

Another emerging indication to perform fecal microbiota transplantation (FMT) is the gastrointestinal (GI) tract colonization by antibiotic-resistant bacteria (ARB), which is, according to the WHO, one of the biggest threats to global health, food security, and development today [197–202]. ARB gut colonization could be detrimental to the patient when causing infections with gut-colonizing organism or, even resulting in immunologic disorders being a marker of gut dysbiosis [203, 204]. Bilinski et al. first reported the high efficacy of FMT reaching 60–100% of different ARB decolonization rate after one month from FMT [198, 205]. The microbiota composition analysis, using next-generation sequencing, showed greater bacterial richness in the donor’s fecal material given to the responders with a higher abundance of *Barnesiella, Butirycimonas and Bacteroides* compared with non-responders. Further studies confirmed these findings [193, 197, 199, 206, 207]. Also, Bilinski’s team have shown the promising results of FMT as an immunomodulatory agent in the treatment of acute gastrointestinal graft-versus-host disease [208, 209] what was confirmed in other groups [210].

Growing evidences show that FMT could be a promising tool for the treatment of both diabetes type 1 and type 2. Indeed, in one of the most recent studies, De Groot et al. demonstrated that FMT can induce a decline in endogenous production of insulin in recently diagnosed patients with T1D 12 months after disease onset. The residual function of beta cells was preserved, and was equally linked to many bacterial strains and microbiota-derived plasma metabolites. Finally, beneficial increase in the whole blood immune cell subsets such as CXCR3 + CD4 + T cells was reported following FMT [211]. Regarding type 2 diabetes, the use of FMT results in the improvement of pancreatic islet β-cells preservation by inhibiting the apoptosis, and in the decrease of insulin resistance. The inflammatory response in the pancreas was decreased, and a decline in the markers of inflammation was noted [212].

A refinement to the FMT, also known as the fecal virome transplantation (FVT), has also been developed. It relies on the use of bacteriophages (phages) to restore the dysbiotic gut microbiota. We previously shed the light on the importance of phages in eliminating the gastroenteritis-associated pathogenic bacteria, and in modulating the beneficial bacteria by adding new functions such as metabolites biosynthesis (SCFAs and H_2_S) in the management of many metabolic and neurological disorders [213]. More recent work, achieved by Hsu et al., revealed that a programmed phage λ can be used to repress *E. coli* genes in the mammalian gut. This virus could be administered in the human body via an oral delivery using an adequate aqueous-based encapsulation formulation [214]. However, despite the promising advantages of FVT, the prophage-encoded virulence factors remain a safety issue, which limits the use of phages in medicine [215].

### Metabolites

The gut microbiota-associated metabolites have not only emerged as pivotal regulators in the development and progression of many human diseases, but also as one of the novel therapeutic strategies. Metabolites are actually used in the treatment of local inflammation and in the modulation of cardiometabolic and neurological disorders, as well as cancers. Their properties make these metabolites relevant therapeutic candidates: natural bioavailability, high concentrations, easy administration, and tissue tolerability [216].

Although the TMAO, an important gut microbe-dependent metabolite, was shown to be involved in the mechanisms of atherosclerotic CVD [217], there is still no consensus about the role of TMAO in the pathogenesis of CVD since regular consumption of TMAO-rich seafood is considered to be beneficial for the primary prevention of cardiovascular events [218]. Indeed, a recent study evidenced that the short-term administration of TMAO did not show any effect on the cardiac functionality. Furthermore, long-term TMAO administration prevented impaired mitochondrial energy metabolism with a tendency of restoring the ventricular function in mice with In-Vitro induced right ventricular dysfunction [219].

In the context of the therapeutic use of metabolites, the SCFAs have been shown to exhibit anti-inflammatory effects, as previously discussed, in modulation of many local and systemic disorders, such in the gut-brain axis [95, 220]. Indeed, the oral administration of SCFAs can alleviate the severe symptoms of brain-associated inflammatory encephalitis [221], and the auto-immune multiple sclerosis [222]. Particularly, ingestion of butyrate not only suppressed the demyelination, but also triggered the remyelination, along with facilitating oligodendrocyte differentiation in mice with induced multiple sclerosis [223]. Furthermore, colon-delivered SCFAs can attenuate hypothalamic–pituitary–adrenal axis reactivity to psychosocial stress [224], suggesting hereby that SCFA supplementation alleviates mood alterations induced by repeated psychosocial stress [225].

Recently, Piscotta et al. investigated the antiviral activity of microbiota derived metabolites on COVID-19. The team found bacterial metabolites that were able to inhibit the infection by COVID-19. These metabolites present similar structural and functional properties to synthetic drugs used for the treatment of COVID-19 patients. The discovered compounds are the nucleoside analogue N6-(Δ2-isopentenyl) adenosine (homologue of remdesivir), the 5-hydroxytryptamine receptor agonist tryptamine (homologous to fluvoxamine), and the pyrazine 2,5-bis(3-indolylmethyl)pyrazine (similar to favipiravir) [226]. Also, Bilinski et al. shown a possible correlation with fecal microbiota transplantation and COVID-19 symptoms relief when FMT administered in the beginning of symptomatic disease [110].

At last, with the advances of metabolomics’ technologies, studies profiling microbiota-derived metabolites have greatly boosted our understanding in the role of the gut-associated metabolites in the cancerogenesis of CRC [227]. Accordingly, new potential clinical applications of such metabolites have arisen in cancer treatment. Given the encouraging results in the preclinical studies, the direct metabolites supplementation has shown success in the CRC prevention and therapy. For instance, butyrate administration, in a short-term clinical study, was able to increase the SCFAs level and prevent the red meat-induced deleterious adduct formation in the rectum [228]. Moreover, butyrate was also shown to enhance the efficacy of radiotherapy in CRC patients [229], suggesting that gut microbiota-derived metabolites could be associated to modalities in the treatment of cancer.

### miRNA

Among all the striking techniques and approaches to manipulate the gut microbiome, some recent findings raise the possibility of using miRNAs as novel therapeutic tools. In fact, microRNAs (miRNA) are short non-coding RNA molecules that regulate gene expression post-transcriptionally. In the intestine, miRNAs are critical for maintenance of homeostasis, and have been known for decades for their role in the posttranscriptional regulation of gene expression at the cellular level. Recently, they have emerged as important regulators of microbiome-host interaction as their role in the inter-species communication has been proven [230]. In human intestines, miRNAs are mainly synthesized in the intestinal epithelial cells and Hopx + cells. Any deficiency in the miRNA synthesis by those cells was associated with gut microbial dysbiosis [230]. Moreover, pre-clinical studies have shown that caecal miRNA signatures depend on the presence of the microbiome, with potential implications for the regulation of the barrier function [231]. In addition, intestinal miRNA may orchestrate responses to pathogenic and probiotic bacteria. For example, colonic miRNA that are deregulated in response to *Citrobacter rodentium* infection are part of a regulatory network affecting apoptosis and hyperproliferation [232]. While some commonalities exist in terms of miRNA responding to different pathogens, specific signatures have been identified. In line with this, probiotic bacteria can alter intestinal miRNA in a species- and strain-specific manner [233]. As an example, *Bifidobacterium bifidum* affects intestinal gene expression via miRNA in a time-dependent manner [233], with implications for probiotic administration protocols. In summary, intestinal miRNAs respond to commensal, pathogenic and probiotic bacteria. Manipulation of the gut microbiome may be a strategy to sustain intestinal health via miRNA.

### Hyaluronan

Hyaluronan (HA) is considered as a novel tool for the development of novel therapeutic agents for the treatment of diseases underlying dysregulation of the microbiota–immune–gut axis [234]. Indeed, in recent years, many researches suggest that HA, an unbranched glycosaminoglycan (GAG) component of the extracellular matrix, may be an effective therapeutics in several pathological conditions [235]. Exogenously administered HA, owing to its remarkable water retention and lubricant properties, has proved to be efficacious in ophthalmic surgeries [236], for the treatment of osteoarthritis [237], and for wound-dressing in ulcers trauma, and burns [238]. Recently, HA appeared to directly modulate the promotion and resolution of IBD by controlling recruitment of immune cells, through the release of inflammatory cytokines, and through balancing homeostasis [239]. The majority of studies on the role of HA in IBD have focused on the involvement of the GAG in the development of fibrotic tissue within the submucosal and muscularis propria layers and on its chemoattractant action for leukocytes in both layers. The biologic effects of HA are mediated by recruiting different receptors, such as CD44 [240], and by promoting the activation of PAMP, such as toll-like receptors, particularly, TLR2 and TLR4, present in different cell types, including fibroblasts, smooth muscle cells, epithelial cells, immune and neuronal cells [241, 242]. During experimental inflammation, HA promotes epithelial repair via TLR4 activation, suggesting the potential therapeutic action of the GAG [243, 244]. Recently, it was demonstrated that, in the rat colon, HA contributes to the formation of an extracellular matrix basal membrane enveloping the surface of myenteric ganglia and of a perineuronal net surrounding myenteric neurons [245]. An experimentally-induced colitis was associated with up-regulation of HA deposition on myenteric ganglia and loss of the HA perineuronal structure, contributing to myenteric neuron derangement during the inflammatory challenge.

### Nanomedicine-based approaches and extracellular vesicles

Novel nanomedicine-based approaches are experimented in the manipulation of the gut microbiota for cancer prevention. Laborious studies are trying to shape nanomaterials able to alter the cancer-causing dysbiotic microorganisms as well as their metabolites found in the cancer microenvironment [246].

The extracellular vesicles, exosomes and their relation with microbiota is now a surging hot topic of interest. In fact, many research groups are now focusing on the communication between microbiota, mitochondria and the host [95]. It was found that microbiota has the ability to interact with host cells and mitochondria, when needed, through extracellular vesicles [247–249]. This interaction could lead to the endocytosis of the extracellular vesicle and its content delivery. The prospective use of microbiota- and host- derived [250] extracellular vesicles in restoring the microbiome integrity and improving mucosal immunity is being extensively reported. Recent findings have shown that exosomal microRNA derived from mesenchymal stem cells plays a strategic role in modulating the gut microbiota and the inflammatory status.

## Conclusion

The gut microbiota evolves with the human evolution. It is also in constant and dynamic interaction with the host gastrointestinal microenvironment, which makes it easily altered by many endogen and external factors. Among the latter, the BE microbiome which can have a great impact on the human health.

We are certain, after multiple impressing and tremendous efforts, that the gut microbiota forms with the host organs a multidirectional crosstalk involved in maintaining a global homeostasis. Owing to the recent scientific advances in this field, these flora-organ interactions are now known as the gut-lung, gut-brain, gut-skin axes, and many others. Any alteration in such axes, in terms of microbial dysbiosis, constitutes one of the main risk factors in the pathogenesis of many diseases including the inflammation of the gastrointestinal tract, metabolic and cardiovascular-renal disorders, as well as neurological disorders. Even if we still have basic understanding in all the underlying mechanisms in such pathologies, a great improvement is achieved in this context, that enables us to think more in depth about targeting the gut microbiota as a treatment. Owing to the advances in molecular biology and sequencing techniques, work is still needed to elucidate more accurately the diversity alteration in the microbial intestinal community leading to the onset or the exacerbation of many diseases. Novel screening or diagnostic tools could be also developed to target more precisely the population at high risk which will enable to prevent such disorders.

In the therapeutic context, targeting specific microbial components or metabolites could provide a tool in the treatment of many diseases. Beyond having the pre- or probiotics, which are the traditional and first line choice of microbial therapies, other strategies are being clinically studied such as the FMT, metabolites, phages and miRNAs.

However, many limitations in the preclinical and clinical findings impede the use of the above strategies in the manipulation of gut microbiota. Hence, more research is required in this field to further explore the considerations that should be addressed while using these strategies to treat patients. Despite all the existing limitations and the discrepancies, targeting the microbiota opens a new therapeutic window for many serious metabolic and neurological disorders, and needs to receive better attention in research due to the hope it provides to many patients. The medicine of tomorrow will come through microbiota. However, our huge challenge is to find subtle and safe strategies to target microbiota dysbiosis to prevent and treat many diseases.

## Data Availability

Not applicable.

## Abbreviations

AD: Alzheimer’s disease
AMR: Antimicrobial-resistance
Aβ: Amyloid beta
BE: Built environment
CDI: *Clostridium difficile* Infections
CDK: Cyclin-Dependent Kinase
CKD: Chronic kidney disease
COPD: Chronic obstructive pulmonary disease
CopEC: Colibactin-Producing *E. coli*
CRC: Colorectal cancer
CVD: Cardiovascular diseases
FMT: Fecal microbial transplantation
FVT: Fecal viral transplant
GAG: Glycosaminoglycan
GDM: Gestational Diabetes Mellitus
GI: Gastrointestinal
H_2_S: Hydrogen Sulfide
HA: Hyaluronan
HAIs: Healthcare-Associated Infections
Hb: Hemoglobin
IBD: Inflammatory Bowel Disease
IL: Interleukin
MDD: Major Depressive Disorder
miRNA: MicroRNA
MMP: Matrix Metalloproteinase
NASH: Non-Alcoholic Steatohepatitis
PAMP: Pathogen-Associated Molecular Patterns
PD: Parkinson's Disease
PE: Pre-eclampsia
rCDIs: Resistant *Clostridium difficile* Infections
SCFAs: Short Chain Fatty Acids
T1D: Type 1 Diabetes
T2D: Type 2 Diabetes
TCAs: T-Tricyclic Antidepressants
TLR: Toll-Like Receptor
TMA: Trimethylamine
TMAO: Trimethylamine N-Oxide
TNF-α: Tumor Necrosis Factor-Alpha
